# Study of assessment of knowledge and understanding for coping with sick days among patients with diabetes in community pharmacy: a cluster randomized controlled trial (SAKURA trial)

**DOI:** 10.1186/s40545-023-00614-4

**Published:** 2023-09-25

**Authors:** Keisuke Kado, Hiroshi Okada, Shota Suzuki, Masako Satake, Toru Yamazaki, Mayumi Kurosawa, Mie Yamamoto, Miho Takahashi, Takeo Nakayama

**Affiliations:** 1 KRAFT Inc. Kyouikukikakubu, Palace Building 10F, 1-1-1 Marunouchi, Chiyoda-ku, Tokyo, 100-8225 Japan; 2 Department of Health Informatics, Graduate School of Medicine and School of Public Health, Kyoto University Yoshida-Konoe-cho, Sakyo-ku, Kyoto, 606-8501 Japan; 3 Institute for Clinical and Translational Science, Nara Medical University Hospital, 840 Shijo-cho, Kashihara, 634-8522 Japan

**Keywords:** Community pharmacy, Diabetes, Education intervention, Implementation research, Randomized controlled trial, Sick day

## Abstract

**Background:**

Awareness regarding coping with sick days among patients with diabetes is limited. Thus, we evaluated the effectiveness of sick-day education by community pharmacists among patients with type 2 diabetes (T2D) using sick-day educational materials (sick-day cards).

**Methods:**

A cluster randomized controlled trial was conducted. Pharmacists in the intervention group educated patients with T2D on coping with sick days (adjusting medication dosage and seeking medical advice) using sick-day cards compared with the usual counseling. Differences in questionnaire scores (“Anxiety”, “Intention”, “Attitude”, and “Knowledge” about sick days) before and after the intervention were compared between the groups.

**Results:**

Overall, 318 patients with T2D (intervention, 119; control, 199) participated in this study, and 270 (intervention, 92; control, 178) patients were examined. There were no significant differences in “Anxiety”, “Intention”, or “Attitude” scores between the two groups, but “Knowledge” scores improved in the intervention group. For all intervention groups (92/92), a physician reviewed and approved medication and adjustment doses for sick days on the cards.

**Conclusions:**

According to patients’ responses, sick-day education using teaching materials improved patient knowledge. This may help patients and their caregivers cope with sick days appropriately through medication dose adjustment and fluid intake.

*Research registration number:* UMIN000043161 (February 1, 2021), https://center6.umin.ac.jp/cgi-open-bin/ctr/ctr.cgifunction=brows&action=brows&recptno=R000048124&type=summary&language=J

## Background

On sick days, during treatment, patients with diabetes experience significant symptoms such as fever, diarrhea, vomiting, or loss of appetite, which may result in life-threatening hyperglycemia and ketoacidosis [1, 2]. Primary care physicians and community pharmacists should provide sick-day education to all patients with diabetes [3]. Most patients with diabetes presenting to the emergency room for severe hypoglycemia or diabetic ketoacidosis (DKA) are older patients with type 2 diabetes (T2D) with an infectious disease characterized by high fever or diarrhea [4]. Therefore, diabetes-related organizations have published various guidelines for patients and medical professionals on coping with sick days [5–9]. In recent years, the use of sodium–glucose cotransporter 2 (SGLT-2) inhibitors has expanded for diabetes treatment, and cases of DKA have been reported even among patients without hyperglycemia [10]. One-third of DKA cases are those with coexisting T2D [11].

The World Health Organization and International Pharmaceutical Federation published a guideline, “Developing Pharmacy Practice: A focus on patient care” in 2000, encouraging pharmacists in the community to shift their focus from traditional dispensing to holistic patient care [12]. A systematic review reported a 0.76% improvement in hemoglobin A1c (HbA1c) levels when pharmacists educated patients with diabetes [13], and various other health promotion interventions have been reported to improve patient outcomes such as blood pressure, asthma, and cholesterol [14, 15]. However, the educational impact of sick days in community pharmacies remains unclear. Moreover, traditional sick-day education is provided by diabetes specialists to adolescents with type 1 diabetes [7, 8]. However, few reports have examined its effectiveness for patients with T2D through a collaboration between pharmacies and primary care physicians.

According to a survey of patients with diabetes and healthcare providers in Japan, 56% of the patients treated with oral medication reported that they were unaware of the sick-day rule, and 66% of the patients reported that they did not receive any guidance regarding sick days [16]. Based on a 2020 survey, the proportion of individuals aged ≥ 65 years has reached 28.4%, which is the highest worldwide [17, 18]. In 2016, the “family pharmacies and pharmacist system” was launched to support the health of residents in this super-aging society. In this system, pharmacies provide various patient care services, such as a 24-h consultation in case of an emergency [19, 20].

We investigated whether education provided to patients by pharmacists, in collaboration with primary care physicians, using sick-day cards about how to deal with sick days, including how to adjust medication dosages and how to see a physician (sick day rules), would change patients' awareness and knowledge of sick days. We also examined the feasibility of pharmacists and physicians working together to educate patients about sick days. Furthermore, we examined the implementation of medical education by physicians and pharmacists and collaboration between them using the RE-AIM (Reach, Effectiveness, Adoption, Implementation, and Maintenance) framework. This framework has been widely used to evaluate the effectiveness of interventions to implement research findings in actual clinical practice [21, 22].

## Methods

### Study design

A randomized controlled trial with clusters of community pharmacies in Japan.

### Setting

Community pharmacies in Japan.

### Participants

#### Cluster level: pharmacy

Pharmacies that accepted more than 50 patients with T2D per month and those whose pharmacists participated in training programs on diabetes care were included.

#### Individual level: patient

Patients with T2D aged ≥ 20 years who were treated with antidiabetic drugs and who recorded their own drug therapy using a medication notebook [23] were included. Japanese patients use Okusuritecho (Medication Notebooks), a small notebook for maintaining their personal medication records. Patients bring their notebooks while visiting their doctors or pharmacies to check their medication.

Patients who were prescribed medication for psychiatric disorders and those facing difficulty (judged by pharmacists) in participating in the survey due to dementia or other illnesses were excluded.

### Randomization

This study was conducted using cluster randomization. After enrollment, eligible pharmacies were stratified and randomized into three strata based on the following requirements considered by the researchers: (1) main medical facilities with a diabetes specialist (with or without); (2) main medical facilities (hospital or clinic) with prescriptions to the pharmacy; and (3) pharmacies with 50-99 and those with > 100 patients with diabetes per month. Allocation personnel not involved in the study assigned each of the 10 pharmacies to one of two groups (Intervention group or Control group) using random number generation in Microsoft Excel®.

### Educational program

#### Training for pharmacists

Pharmacists who agreed to participate in this study underwent briefing and training sessions by the researchers.

1. Briefing session for conducting the study: Overview of the study and questionnaire survey method (1 h).

A briefing session was conducted using a web conferencing system (WebEx®). Pharmacists who were unable to attend the briefing session watched a video recording of the briefing at a later date, and the researchers confirmed their understanding. In the briefing session, the outline of the research plan, its purpose and significance, the procedure for conducting the questionnaire survey, and willingness to participate in the research were confirmed.

2. Training on sick days for pharmacists: How to use the sick-day card (3 h) (Table 1).

**Table 1:** Recruitment briefing for study participation and training

Time (min)	Recruitment and explanation
10	Introduction
40	Study purpose and protocol
10	Informed consent
Total 60	

Time (min)	Training for sick-day education
10	Introduction
40	Study flow and procedure in this survey
40	Lecture: How to use the sick-day card
90	Role-playing and discussion
Total 180	

After the briefing, the pharmacists assigned to the intervention group were trained on another day regarding patient education on sick-day countermeasures using the web conference system. Pharmacists who were unable to attend the training session watched a video recording of the training at a later date. In addition, the role-play part of the session was tutored by the instructor. The training consisted of two parts:

A lecture on the basic knowledge and symptoms of diabetes based on the Japanese Diabetes Association and Japan Pharmaceutical Diabetes Society guidelines [1, 24], including lectures on hypoglycemia, sick days, and how to use the sick-day card. This was followed by a discussion among the participants, where the points to be considered during the questionnaire survey were summarized, such as posture and talking to the patient.

Participants were divided into two groups, pharmacist group and patient group, and role-playing was used to explain sick days.

#### Sick-day card

**Fig. 1:**
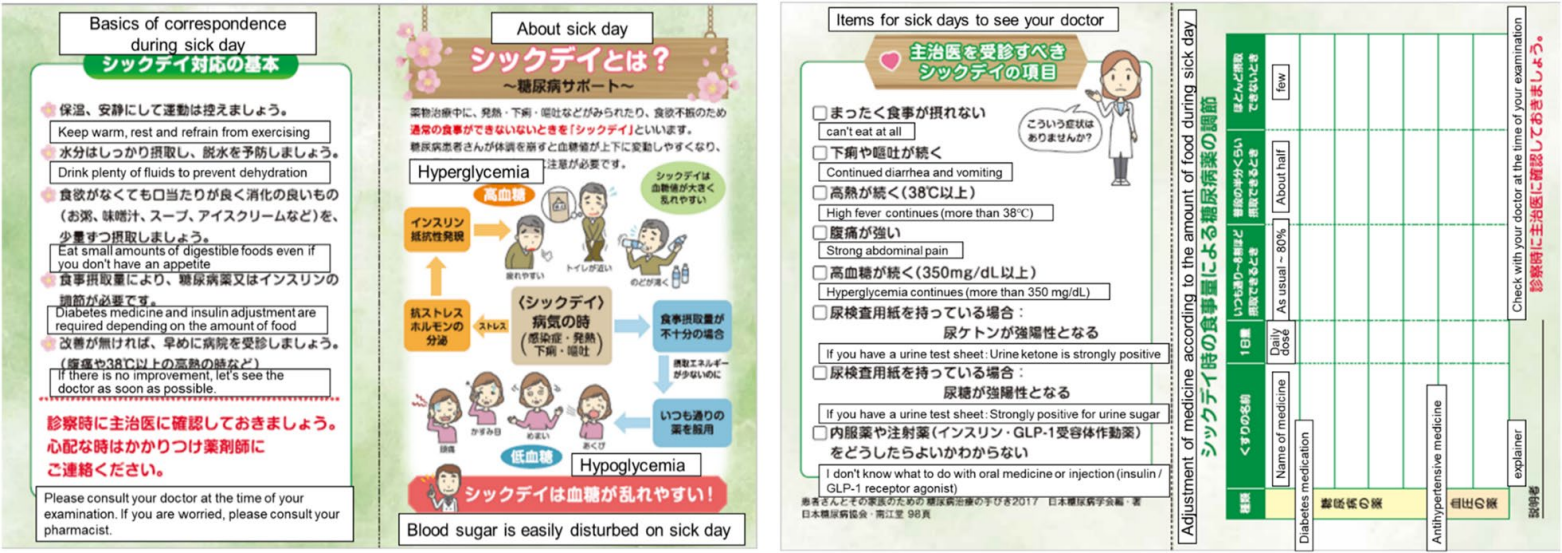
Sick-day card. Reproduced with permission from KRAFT Inc

The sick-day card is a piece of educational material the size of a medication notebook (A5 size), which can be inserted into the medication handbook. It was developed by a pharmacist (MS) as a prototype in accordance with the Diabetes Care Guidelines [1, 24] and was completed under the supervision of a diabetes specialist (MT) to help community pharmacists promptly and accurately inform patients with diabetes about sick-day rules. The card describes medication dosage adjustments and when medical help is required; it has a column for pharmacists/primary care physicians to fill in medication details, highlighting any discontinuation or change during sick days (Fig. 1).

### Intervention (patient education)

While administering medication to patients with diabetes, the pharmacists explained the minimum required information needed to cope with sick days on the sick-day card. The explanation answered the following items: (1) “What is a sick day?” (2) “Item for sick days to see your physician” and (3) “What are the adjustments of medications based on dietary intake?” For each type of diabetic medication, the specific dosage according to food consumption (as usual, approximately 80%, about half, or few) was indicated.

After the explanation, the card was fastened to the medication notebook with a pink rubber band, and the patients were instructed to confirm and sign the diabetes medication adjustment with their physician at their next visit. The next time the patients visited the pharmacy, the physician’s authorization on the card was assessed, and the questionnaire was re-administered.

Community pharmacists in the control group provided usual medication instructions to the patients. Patients were instructed to complete the questionnaire only at the time of prescription receipt. The next time the patients visited the pharmacy, the same questionnaire was re-administered. In the case of refusal to participate in the study, normal medication instruction was provided, and after the study period, education using a sick-day card was provided to avoid disadvantages.

### Outcomes

For the first survey, all eligible patients who received prescription medicines at the pharmacy during the recruitment period were instructed to cooperate (baseline). The second questionnaire was administered 1–3 months after the first survey, when the patients revisited the pharmacy after appointment with a physician.

#### Primary outcome

The primary outcome was the difference in sick-day scores of patients between the two groups from baseline to the next visit (1–3 months later).

#### Sick-day scores

Since there are no previous studies of sick-day education for patients with T2D patients in pharmacies, the items of the questionnaire in this study were developed through repeated discussions between public health researchers and pharmacists in the field involved in diabetes care. Each score comprised two questions, and each question was answered on a six-point Likert scale. The patient’s “Anxiety”, “Intention”, and “Attitude” regarding “medication dosage adjustment” and “decision to see a doctor” during sick days were scored, and these scores were used to indicate “understanding”. Higher scores indicated higher anxiety, stronger intentions, and more positive attitudes.

The “Knowledge” questionnaire consisted of eight questions to assess the patient’s knowledge about sick days. Participants answered “true”, “false”, or “I do not know” (scored as incorrect), and the final score comprised the total number of correct answers (0–8 points). Regarding patient education using the sick-day card, the number of patients in the intervention group who had their card approved by the physician and who brought their cards to their next visit were investigated. After the study was completed, the participating pharmacists were interviewed about the time required to educate each patient.

### Sample size and statistical analyses

When the difference between the mean scores from the pre-intervention and post-intervention questionnaires for each item was set at 1.0, standard deviation (SD) was 4.0, alpha error was 0.05, power was 0.8, and the required number of patients was approximately 500. The sample size was estimated to be approximately 550 patients, accounting for approximately 10% dropout rate during the study. Fifty to hundred patients were assumed to visit each pharmacy per month; hence, the number of pharmacies was set to 10.

Data analysis was performed using EZR (Jichi Medical University, Saitama, Japan) [25], and a *p*-value < 0.05 was considered statistically significant. The difference in the scores from the questionnaires administered before and after the intervention between the two groups was analyzed using the Mann–Whitney U test, and “Anxiety”, “Intention”, “Attitude”, and “Knowledge” scores were analyzed using the t-test without correspondence.

### Informed consent

Written consent was obtained from pharmacists working at the pharmacies, and participating pharmacies displayed posters with information about the study. Patients visiting the pharmacies were asked to verbally state their intention to cooperate, and their consent was recorded.

## Results

Education using sick-day cards was provided to patients with T2D in pharmacies in cooperation with their physicians. Patients’ “Knowledge” and “Attitude” scores tended to improve after education by pharmacists.

### Study period

The study period was between April 1, 2021, and December 31, 2021; patients were enrolled from the beginning of the study until April 30, 2021.

### Participants

Twelve pharmacies wanted to participate in this study. However, two pharmacies withdrew because they could not obtain consent from neighboring medical institutions to cooperate; thus, this study was conducted in ten pharmacies. Of these, six pharmacies received prescriptions from clinics and four received them from hospitals; two of the four hospitals had diabetes specialists (Table 2).

**Table 2:** Baseline characteristics of individuals and clusters

	Intervention group	Control group
*Cluster level*		
No. of pharmacies	5	5
Cluster type		
Pharmacies by the hospital^*^		
Diabetes specialist	1	1
No diabetes specialist	1	1
Pharmacies by the clinic^†^		
Small (50–99)	2	2
Large (> 100)	1	1
*Individual level*		
No. of patients	92	178
Mean (SD) age (years)	67.1 (10.5)	66.1 (11.7)
Sex Male/female/unknown	49/43/0	113/60/5
Diabetes medication (%)		
Sulphonylurea	21/92 (22.8)	29/173 (16.8)
Glinide	5/92 (5.4)	22/173 (12.7)
α-GI	16/92 (17.4)	32/173 (18.5)
Biguanide	44/92 (47.8)	88/173 (50.9)
Thiazolidine	5/92 (5.4)	16/173 (9.2)
DPP-4	57/92 (62.0)	122/173 (70.5)
SGLT2	26/92 (28.3)	81/173 (46.8)
GLP-1	5/92 (5.4)	6/173 (3.5)
Insulin	10/92 (10.9)	11/173 (6.4)

^*^Hospitals were categorized by the presence or absence of a diabetes specialist

^†^Clinics were categorized by the number of diabetic patients visiting pharmacies next to the clinic a month. Small 50–99, large > 100

*SD* standard deviation

*α-GI* α-glucosidase inhibitor, *DPP-4* dipeptidyl peptidase 4 inhibitor, *SGLT2* sodium–glucose cotransporter 2 inhibitor, *GLP-1* glucagon-like peptide 1 agonist

#### Patients with type 2 diabetes

The mean ages of the 270 patients in this study were 67.1 (SD, 10.5) and 66.1 (SD, 11.7) years in the intervention and control groups, respectively. The male/female ratios were 49/43 and 113/60 (with 5 unknowns) in the intervention and control groups, respectively.

### Implementation outcomes: RE-AIM (Reach, Effectiveness, Adoption, Implementation, and Maintenance) framework

#### Reach

**Fig. 2:**
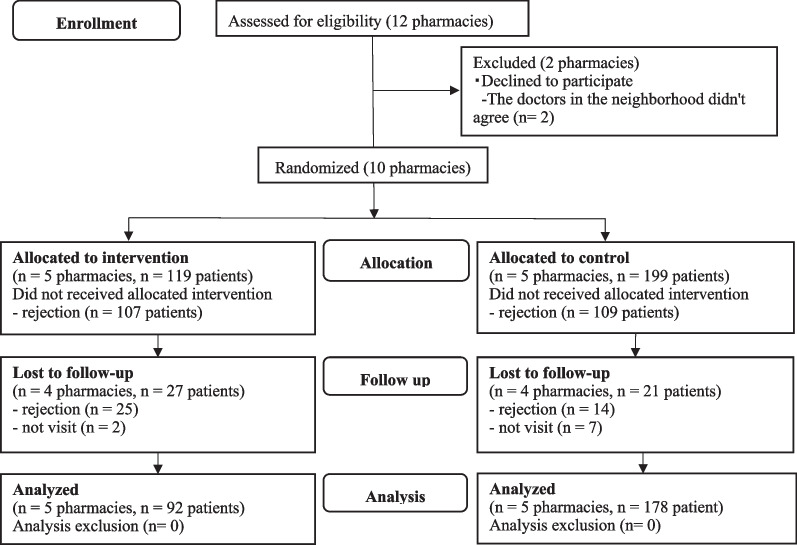
Consolidated Standards of Reporting Trials (CONSORT) flowchart of recruitment in the SAKURA study

During the study period, 554 patients with T2D visited the participating pharmacies (intervention: 227, control: 327), and 20 of them violated the exclusion criteria (intervention: 1, control: 19). Therefore, 534 patients (intervention: 226, control: 308) were included in the study, of whom 318 (intervention, 52.7% [119/226]; control, 64.6% [199/308]) consented to the first questionnaire survey. One to three months later, 270 participants responded to the second survey (intervention, 40.7% [92/226]; control, 57.8% [178/308]) (Fig. 2).

#### Effectiveness

There was no significant difference between the two groups in the scores of “Anxiety” about sick days and “Intention” to respond to the questionnaire, from baseline to the next visit. The difference in “Attitude” score was 0.7 (95% confidence interval [CI], -0.2 to 1.4; *p* = 0.13). The difference in “Knowledge” score was 1.0 (95% CI, 0.4 to 1.5; p < 0.01) (Table 3, Appendix Table 4). The change in satisfaction with the pharmacist was -0.1 (4.8) in the control group, but it improved to 0.8 (7.7) in the intervention group, with a difference of 0.9 (95% CI, -0.7 to 2.3; *p* = 0.29) between the two groups. Intra-class correlations (ICC) based on mixed model analysis are presented in Appendix Table 5 to indicate clustering effects for the investigated outcomes.

**Table 3:** Scores on sick-day Anxiety, Intention, Attitude, and Knowledge

	Intervention group (*n* = 92) mean (SD)			Control group (*n* = 178) mean (SD)			Intervention vs. control	
(/ full-score points)	Pre	Post	Difference	Pre	Post	Difference	Difference (95% CI)	*p*-value^**^
Anxiety score^*^ (/12)	5.2 (2.6)	5.3 (2.1)	0.1 (2.8)	5.4 (2.4)	5.5 (2.3)	0.1 (2.6)	0.0 (− 0.6 to 0.8)	0.79
Intention score^†^ (/12)	6.7 (3.1)	6.5 (2.5)	− 0.2 (3.4)	6.4 (2.5)	6.5 (2.4)	0.1 (2.5)	− 0.3 (− 1.0 to 0.4)	0.39
Attitude score^§^ (/12)	7.7 (3.1)	8.2 (2.6)	0.5 (3.8)	7.9 (2.7)	7.7 (2.5)	− 0.2 (2.7)	0.7 (− 0.2 to 1.4)	0.13
Pharmacy satisfaction score^||^ (/36)	22.0 (6.7)	22.8 (6.0)	0.8 (7.7)	21.9 (4.9)	21.8 (5.0)	− 0.1 (4.8)	0.9 (− 0.7 to 2.3)	0.29
Knowledge score^¶^ (/8)	2.1 (2.0)	3.5 (2.6)	1.4 (2.9)	2.0 (2.0)	2.4 (2.2)	0.4 (1.8)	1.0 (0.4–1.5)	< 0.01

^*^Anxiety score = total score of six-point Likert scale on two items

^†^Intention score = total score of six-point Likert scale on two items

^§^Attitude score = total score of six-point Likert scale on three items

^||^Pharmacy satisfaction score = total score of six items

^¶^Knowledge score = the number of correct answers to eight true/false quizzes about sick day

^**^P-value for comparison of the difference in values between the intervention and control groups (Mann–Whitney U test, Student’s t-test)

#### Adoption

Of the 119 patients in the intervention group who agreed to participate in the first questionnaire survey, two did not return for the next visit and 25 refused to participate in the second survey, resulting in 40.7% (92/226) having results of both surveys. Out of the 199 cooperators in the control group, seven failed to follow-up the second time and 14 refused to cooperate for the second time, finally resulting in 57.8% (178/308) of the participants.

#### Implementation

The cost of producing a sick-day card was approximately 4 yen (3.5 cents). Pharmacists reported that it took 3–4 min to educate patients about sick days using sick-day cards in their pharmacy.

#### Maintenance

The attending physician reviewed the sick-day cards of all patients (92 patients in the intervention group) and approved the pharmacists’ entries on the card regarding the patients’ dosage during sick days. All patients brought their sick-day cards to their next clinic visit and continued to use them.

The results of each question and item in the survey are shown in the Appendix Table 6.

## Discussion

In this study, pharmacists provided information about sick days to the intervention group of patients with T2D using sick-day cards at the time of medication administration. No statistically significant differences in the sick-day scores between the intervention and control groups were found for “Anxiety”, “Intention”, and “Attitude”. For “Anxiety” score, the baseline pre-intervention value was low (intervention, 5.2 [2.6]; control, 5.4 [2.4]), which may have prevented the detection of a difference in the educational effect. Previous studies have reported low awareness of sick days among patients with diabetes [15], and the baseline “Anxiety” score might be lower than expected because many patients are unaware of the risks associated with sick days. Additionally, even if a patient with T2D experiences a sick day, it often causes no serious problems that might require emergency medical care, except in the elderly. Therefore, many patients may be less likely to realize that a sick day is a serious threat to their life; thus, they are not concerned about adjusting their medications. Regarding the effectiveness of sick-day education in this study, the scores for “Intention” and “Attitude” may also have been lower than expected. Patients with diabetes who have not experienced difficulty while coping with a sick day and are unaware of its life-threatening consequences may not realize the benefit of this intervention. We believe this is supported by the fact that the percentage of patients who agreed to participate was lower in the intervention group than in the control group (intervention, 52.7% [119/226] vs. control, 64.6% [199/308]). Patients unaware of sick days and crises may be less likely to perceive the benefits of education from pharmacists about sick days and may have experienced less change in their “Intention” and “Attitude” toward dealing with sick days, compared with those who are aware. Regarding the influence of the clusters, we judged that the effect of the clusters on the results was limited even when the ICC was considered, given that this was a questionnaire survey and the ICC was low at < 0.1.

Patients’ knowledge of sick days begins at hospitalization or during outpatient care, when doctors, nurses, and pharmacists elaborate on sick days and the rules of changing medication dosage when food intake is reduced [9]. However, many patients do not remember learning about sick days because they cannot visualize the situation or believe that they will never face such a situation [15]. The sick-day card in this study could be used to reduce the amount of medication in each patient’s prescription. Therefore, even if the patient does not remember the healthcare provider’s explanation, the card can be inserted into the medication notebook so that the patient can refer to it and obtain information on the amount of medication reduction required for sick days. A sick-day card is a reference for patients with T2D in an emergency situation to help them adjust their medication dosage and/or decide whether to consult a doctor, regardless of their existing knowledge of sick days. The card may also be a guide for diabetes medication according to the rationed food supply in emergency situations.

In a few studies, pharmacists collaborated with physicians to provide sick-day education to patients with diabetes, and most previous studies have focused on adolescents with type 1 diabetes [7, 8]. In the present study, the pharmacists proposed adjustments to the medications listed on the sick-day cards for patients with T2D, including oral medications and insulin, and a physician confirmed and approved all 92 patients in the intervention group. For the two insulin-treated patients, adjustment of the injection dose was added to their cards. The physicians utilized the cards this way probably because they understood study and approved the use of sick-day cards; moreover, the patients were instructed to always show their cards to the physicians. However, the most important reason for using the system in Japanese medical institutions is that checking the medications listed in the medication handbook is part of the workflow of doctors and pharmacists. Therefore, inserting a sick-day card into the handbook enables the doctors to check the medications. Using the sick-day card to provide education, including drug adjustment for sick days, may provide opportunities for education and for sharing information on patients with diabetes between physicians and pharmacists.

The limitation of this study was the patients’ questionnaire survey because it did not clarify actual patient management for sick days. To examine the actual educational effect, it would be necessary to examine the difference in the incidence of severe hypoglycemia and ketoacidosis, which are associated with sick days. However, we did not adopt this validation method for this study because the incidence occurs rarely with a long follow-up period. Generally, sick-day education in pharmacies would lead to a violation of physician discretion because it would reduce the use of medications. Therefore, sick-day cards were created, and information is shared with physicians. In addition to the fact that the facilities that participated in this study were considered more committed to patient education than they are to other general practices or hospitals, the data of individuals who refused to participate in the study were not included. Unfortunately, this study did not investigate the explicit reasons for refusal, and this could be because pharmacies have a relationship with pharmacists that makes it easy for them to reject their offers and because they do not have the time to participate in the study, which may be the possible reasons for the high estimate of the educational effect in this study. The strength of this study is that the web program for sick-day education for pharmacists was relatively short, i.e., it lasted for only 180 min; thus, it can be operated efficiently and cheaply online using the videos. The use of sick-day cards in pharmacies has great potential for social implementation because education can be provided in a matter of minutes. These suggest that collaboration between physicians and pharmacists using sick-day cards is a feasible way to educate the public about sick days.

This study suggests that sick-day cards can be used to educate patients with diabetes in pharmacies. We plan to follow up with patients to determine the extent to which they are able to utilize these cards to address their sick days’ needs. Furthermore, in 2022, the Japan Drug and Diabetes Association will begin promoting their use to help patients manage their sick days [26]. We would like to investigate whether there is a difference in the emergency transport of patients with diabetes between areas where the cards are distributed and where they are not.

Conclusion.

Using the sick-day cards, community pharmacists improved knowledge and provided clarity regarding medication adjustment among individual patients on sick days. A simple web-based educational program was also implemented on sick days for community pharmacists, in collaboration with physicians, to provide education without spending time and money. The use of sick-day cards can clarify the sick-day rule, which is highly individualized for the patients, and it may be useful for preventing serious diseases that affect prognosis, by promoting social implementation.

## Data Availability

The anonymized datasets used and/or analyzed during the study are available from the corresponding author upon reasonable request.

## Appendix

See Tables 4, 5 and 6

**Table 4:** Questions about Sick Day Knowledge

	Intervention group (*n* = 92), correct answers			Control group (*n* = 178), correct answers			Intervention vs. control	
Questions about Sick Day Knowledge Items	Pre	Post	Difference	Pre	Post	Difference	Difference	*p*-value^**^
Does stress lower blood glucose levels during a sick day?	8	20	12	20	26	6	6	0.23
On a sick day, is blood sugar disrupted because of loss of appetite and inability to eat?	24	44	20	45	59	14	6	0.34
Does anyone take the usual amount of medication during a sick day?	14	43	29	40	59	19	10	0.06
Do you withhold fluids during a sick day to prevent diarrhea?	34	49	15	62	78	16	-1	0.68
Does high blood sugar usually cause symptoms such as headache, blurred vision, dizziness, or yawning?	12	27	15	17	16	-1	16	0.094
Does low blood sugar usually make you tired, thirsty, or close to the bathroom?	20	33	13	22	23	1	12	0.31
If I have a sick day and don't have an appetite, should I not eat?	32	39	7	47	60	13	-6	1
Should a patient be seen as soon as possible during a sick day when the fever is higher than 38℃?	50	70	20	100	115	15	5	0.42

^*^*P*-value for comparison of the difference in values between the intervention and control groups (Chi-square test)

**Table 5:** Intra-class coefficients (ICC) for clustering effects pre (intervention *N* = 92; control *N* = 178) and post (intervention *N* = 92; control *N* = 178) education

	Pre	Post
Anxiety score^*^ (/12)	0.04	0.07
Intention score^†^ (/12)	0.07	0.06
Attitude score^§^ (/12)	0.02	0.05
Pharmacy satisfaction score^||^ (/36)	0.03	0.05
Knowledge score^¶^ (/8)	0.05	0.08

^*^Anxiety score = total score of six-point Likert scale on two items

^†^Intention score = total score of six-point Likert scale on two items

^§^Attitude score = total score of six-point Likert scale on three items

^||^Pharmacy satisfaction score = total score of six items

^¶^Knowledge score = the number of correct answers to eight true/false quizzes about sick day

**Table 6:** Sick-day questionnaire items

	Intervention group (*n* = 92), mean (SD)			Control group (*n* = 178), mean (SD)			Intervention vs. control	
Sick-day questionnaire items	Pre	Post	Difference	Pre	Post	Difference	Difference (95% CI)	*p*-value^**^
During sick days								
Are you anxious about adjusting your medication?	2.3 (1.3)	2.5 (1.2)	0.2 (1.6)	2.4 (1.3)	2.5 (1.2)	0.1 (1.5)	0.1 (− 0.3 to 0.5)	0.60
Are you anxious about when to see a doctor?	2.9 (1.5)	2.8 (1.3)	− 0.1 (1.7)	3.1 (1.5)	3.0 (1.4)	− 0.1 (1.6)	0.0 (− 0.5 to 0.4)	0.93
Do you know how to adjust your medication?	3.3 (1.7)	3.2 (1.4)	− 0.1 (2.0)	3.0 (1.4)	3.1 (1.3)	0.1 (1.6)	− 0.2 (− 0.7 to 0.2)	0.43
Do you want to know when to see a doctor?	3.4 (1.7)	3.3 (1.5)	− 0.1 (1.8)	3.4 (1.5)	3.4 (1.4)	0.0 (1.5)	− 0.1 (− 0.5 to 0.3)	0.72
If you know the drug adjustment instructions, can you adjust it?	4.0 (1.6)	4.2 (1.4)	0.2 (2.1)	4.0 (1.5)	3.9 (1.4)	− 0.1 (1.7)	0.3 (− 0.2 to 0.7)	0.48
If you have symptoms would you see a doctor?	3.7 (1.7)	4.0 (1.5)	0.3 (2.1)	3.9 (1.5)	3.8 (1.4)	− 0.1 (1.5)	0.4 (− 0.1 to 0.8)	0.23
I expect explanations that are in line with my treatment policy and lifestyle At this pharmacy	4.0 (1.5)	4.1 (1.3)	0.1 (1.8)	3.8 (1.2)	3.9 (1.1)	0.1 (1.2)	0.0 (− 0.4 to 0.3)	0.85
Satisfied with providing information about diabetes	4.1 (1.4)	4.3 (1.1)	0.2 (1.8)	4.0 (1.1)	3.9 (1.0)	− 0.1 (1.4)	0.3 (− 0.1 to 0.7)	0.16
I want to talk about illnesses other than diabetes	3.6 (1.5)	3.5 (1.4)	–0.1 (1.8)	3.5 (1.2)	3.4 (1.2)	− 0.1 (1.4)	0.0 (− 0.4 to 0.4)	0.86
I want to talk about meals	3.2 (1.5)	3.4 (1.3)	0.2 (1.5)	3.3 (1.2)	3.3 (1.1)	0.0 (1.0)	0.2 (− 0.1 to 0.5)	0.27
I want to talk about exercise	3.1 (1.5)	3.4 (1.2)	0.3 (1.5)	3.2 (1.1)	3.2 (1.1)	0.0 (1.0)	0.3 (0–0.6)	0.048
I can recommend this pharmacy to others	4.0 (1.5)	4.1 (1.3)	0.1 (1.5)	4.2 (1.2)	4.2 (1.0)	0.0 (1.0)	0.1 (− 0.2 to 0.4)	0.45

^*^*P*-value for comparison of the difference in values between the intervention and control groups (Mann–Whitney *U* test, Student’s *t*-test)

## Abbreviations

CI: Confidence interval
DKA: Diabetic ketoacidosis
T2D: Type 2 diabetes

## References

[1] Araki E, Goto A, Kondo T, Noda M, Noto H, Origasa H (2020). Japanese clinical practice guideline for diabetes 2019. Diabetol Int.

[2] McDermot PA (1998). Diabetic emergencies and sick day rules. Home Care Provid.

[3] Eledrisi MS, Elzouki AN (2020). Management of diabetic ketoacidosis in adults: a narrative review. Saudi J Med Med Sci.

[4] Guisado-Vasco P, Cano-Megías M, Carrasco-de la Fuente M, Corres-González J, Matei AM, González-Albarrán O. Clinical features, mortality, hospital admission, and length of stay of a cohort of adult patients with diabetic ketoacidosis attending the emergency room of a tertiary hospital in Spain. Endocrinol Nutr. 2015;62:277–84. 10.1016/j.endonu.2015.02.003 25888157

[5] American Diabetes Association. Treatment & care, preparing for sick-days. https://www.diabetes.org/diabetes/treatment-care/planning-Sick-days. Accessed 14 Mar 2022.

[6] International Diabetes Federation. How to manage diabetes during an illness? Sick-day rules. https://www.idf.org. Accessed 14 Mar 2022.

[7] Laffel LM, Limbert C, Phelan H, Virmani A, Wood J, Hofer SE (2018). ISPAD Clinical Practice Consensus Guidelines 2018: sick day management in children and adolescents with diabetes. Pediatr Diabetes.

[8] Pichert JW, Snyder GM, Kinzer CK, Boswell EJ (1994). Problem solving anchored instruction about sick days for adolescents with diabetes. Patient Educ Couns.

[9] Association of Diabetes Care & Education Specialist. https://www.diabeteseducator.org/docs/default-source/education-and-career/Sick-day_adult.pdf?sfvrsn=2. Accessed 14 Mar 2022.

[10] Modi A, Agrawal A, Morgan F (2017). Euglycemic diabetic ketoacidosis: a review. Curr Diabetes Rev.

[11] Musso G, Saba F, Cassader M, Gambino R (2020). Diabetic ketoacidosis with SGLT2 inhibitors. BMJ.

[12] Wiedenmayer K, Summers RS, Mackie CA, Gous AGS, Everard M. Developing pharmacy practice: a focus on patient care [Handbook]. 2006. https://apps.who.int/iris/handle/10665/69399. Accessed 14 March 2022.

[13] Collins C, Limone BL, Scholle JM, Coleman CI (2011). Effect of pharmacist intervention on glycemic control in diabetes. Diabetes Res Clin Pract.

[14] Hazen ACM, de Bont AA, Boelman L, Zwart DL, De Gier JJ, De Wit NJ (2018). The degree of integration of non-dispensing pharmacists in primary care practice and the impact on health outcomes: a systematic review. Res Social Adm Pharm.

[15] Steed L, Sohanpal R, Todd A, Madurasinghe VW, Rivas C, Edwards EA (2019). Community pharmacy interventions for health promotion: effects on professional practice and health outcomes. Cochrane Database Syst Rev..

[16] Diabetes net. [Tonyobyou Network in Japanese] 6% of people with diabetes experience sick-days, but their awareness is low. [Tonyobyou-Kanjasanno 6wariga shikkudei wo taikensurumo Ninchihikui]. 2012. https://dm-net.co.jp/calendar/2012/018565.php. Accessed 14 Mar 2022.

[17] Japan Cabinet Office [internet]. Annual report on the aging society FY2020 [cited 14 Mar 2022]. Available from: https://www8.cao.go.jp/kourei/english/annualreport/2020/pdf/2020.pdf.

[18] Organization for Economic Co-operation and Development (OECD). Elderly population. https://data.oecd.org/pop/elderly-population.htm. Accessed 14 Mar 2022.

[19] Japan Ministry of Health, Labour and Welfare. Information on pharmacies and pharmacists [Yakkyoku, Yakuzaisi ni kansurujouho]. https://www.mhlw.go.jp/stf/seisakunitsuite/bunya/kenkou_iryou/iyakuhin/yakkyoku_yakuzai/index.html. Accessed 14 Mar 2022.

[20] Tachi T, Noguchi Y, Teramachi H (2020). Developing and verifying the efficacy of “Educational Program for Promoting Appropriate Self-Medication via Pharmacies and Pharmacists”: a randomized controlled trial. Biol Pharm Bull.

[21] Glasgow RE, Vogt TM, Boles SM (1999). Evaluating the public health impact of health promotion interventions: the RE-AIM framework. Am J Public Health.

[22] RE-AIM. https://re-aim.org/. Accessed 14 Mar 2022.

[23] Onda M, Kuwanoe T, Hashimoto A, Horiguchi M, Domichi M, Sakane N (2021). Pharmacist-delivered Smoking Cessation program in community pharmacy (the FINE program) in Japan-the development of a training course and a feasibility study. J Pharm Pract.

[24] Japan Pharmaceutical Association, Japan Pharmaceutical Diabetes Society. Diabetes care guideline by pharmacist [Yakuzaishiniyoru Tounyouby-shinryo Guideline in Japanese]. Tokyo: Jiho; 2018.

[25] Kanda Y (2013). Investigation of the freely available easy-to-use software “EZR” for medical statistics. Bone Marrow Transplant.

[26] Japan Pharmaceutical Diabetes Society. https://jpds.or.jp/. Accessed 14 Mar 2022

